# Comparative Study of Antibacterial Effects and Bacterial Retentivity of Wound Dressings

**Published:** 2013-01-24

**Authors:** Toshihiro Fujiwara, Ko Hosokawa, Tateki Kubo

**Affiliations:** ^a^Department of Plastic Surgery, Osaka University Graduate School of Medicine; ^b^Department of Plastic Surgery, Osaka Rosai Hospital, Osaka, Japan

## Abstract

**Objectives:** We are often confused on selecting a suitable wound dressing for the treatment of infected wounds from huge number of available wound dressings. Then, to help clinicians easily select a wound dressing, we compared the antibacterial effects and bacterial retentivity (ie, potency of keeping absorbed bacteria inside wound dressings and preventing them from leaking out) of wound dressings. **Methods:** Five wound dressings with antibacterial constituents were compared to research antibacterial effects against nonpathogenic *Escherichia coli* using an in vitro model. The 5 other wound dressings with no antibacterial constituent were compared to research bacterial retentivity. The relative amount of *E coli* was determined using cell proliferation reagent WST-1 (11644807001, Roche Applied Science, United States) with time. **Results:** The results have shown that the antibacterial effects and bacterial retentivity differed among various wound dressings. Silver ions quickly exerted a very strong antibacterial effect, and hydrofibers had a high potency of bacterial retentivity by gelling the absorbed bacteria in wound dressings. **Conclusions:** The present study indicated the differences of antibacterial strength, time of onset and duration of the antibacterial effect, and bacterial retentivity between each wound dressing. Clinicians should use appropriate wound dressings according the wound condition in consideration of the different characteristics of wound dressings. The present results are helpful for clinicians to select appropriate wound dressing.

In 1962, the concept of moisture wound healing has been advocated.^1^ Since then, materials of wound dressing have been also developed to absorb plenty of exudate and keep moisture on wound surface.^2^^,^^3^ Major materials, which are often used in the clinical practice, include hydrocolloids, polyurethane foams, alginates, hydrogels, and hydropolymer.^4^ The absorption ability of each wound dressing is different. Then, we select a suitable wound dressing after assessment of the wound condition; for example, blood flow, degree of moisture on the wound surface, amount of exudate, and infection. Especially in a case of chronic wound, we must carefully select a wound dressing because the condition of chronic cutaneous ulcer varies among patients.^5^^,^^6^ Moreover, various types of bacteria exist on a chronic wound surface and inside chronic wound, resulting in delaying wound healing.^7^ Warmth, humidity, and nutrient-rich soil are necessary for proliferation of bacteria.^8^ Wet wound dressings may provide optimal condition not only for the healthy cells in the wound but also for bacteria.

To treat an infected wound, a gauze dressing coated with an antibacterial constituent is often used.^9^ However, the gauze dressing cannot keep optimal moisture environment on the wound surface and absorb plenty of exudate. Therefore, wound dressing including antibacterial effect, not only moisture effect, has been developed. We are often confused on selecting a suitable wound dressing for infected wounds because the types of wound dressings are too much. Factors involved in selecting wound dressings include dressing properties, wound condition, cost, easiness, and adverse effects.^10^ The antibacterial effect is also an important factor on selecting a wound dressing for management of infected wound. We previously investigated the antibacterial penetrating effects of wound dressings^11^ and reported that each wound dressing has different penetrating effects. The results help clinicians select from a variety of wound dressings.

In the present study, we compared the antibacterial effects of major wound dressings to help clinicians easily select a wound dressing. Next, we compared the “bacterial retentivity” of wound dressings as another antibacterial effect. Wound dressings include a retentivity potency to keep absorbed bacteria inside the wound dressing and prevent them from leaking out the wound dressings. Keeping absorbed bacteria inside wound dressing is also one of the antibacterial effects because the bacterial retentivity contributes to reduction of bacteria on the wound surface. Then, we developed an in vitro model using *Escherichia coli* as a bacterium and investigated the antibacterial effects and bacterial retentivity of each wound dressing.

## METHODS

### Antibacterial effects of wound dressings

#### Preparation of different wound dressings

To compare the antibacterial effects of wound dressings used in the clinical practice, we prepared 0.1% gentamicin sulfate (Gentacin ointment, Schering-Plough, Japan)-coated gauze, hydrofiber with 1 parts per million of ionic silver (Aquacel Ag, ConvaTec, United States), 1% sulfadiazine silver (Geben cream, Mitsubishi Tanabe Pharma Corporation, Japan)-coated gauze, sucrose with 3% povidone-iodine (U-pasta ointment, Kowa, Japan)-coated gauze, and iodoform gauze (Tamagawa, Japan), of which each sheet contains 1.1 g iodoform per 0.3 m^2^. No wound dressing was regarded as a control. Next, each wound dressing or gauze was cut into squares 1 cm^2^ in size. Each piece of wound dressings was put in a well of a 24-well plate.

#### Preparation of bacteria

We used nonpathogenic *E coli* to investigate the antibacterial effects of each wound dressing. *E coli* was cultured with Luria-Bertani (LB) liquid medium at 37°C until confluent growth and diluted to 0.01% with the antiseptic LB liquid medium. Next, 2 mL of the diluted LB liquid medium with *E coli* were gently poured into a well containing a piece of the respective wound dressing. Then, *E coli* in the 24-well plate was cultured in an incubator at 37°C for 24 hours.

#### Determination of the bacterial amount

Initially, 100 μL of the LB liquid medium with *E coli* was collected from each well with wound dressings at 1, 5, 15, 30 minutes and at 1, 3, 6, 12, 24 hours after starting the culture and poured in a 96-well microplate. Then, 10 μL of cell proliferation reagent WST-1 (11644807001, Roche Applied Science, United States) was added to each well of the 96-well microplate and mixed slightly. The LB liquid medium with WST-1 was incubated at 37°C for 2 hours. Next, absorbance at 450 nm and 650 nm was determined using a microplate reader (iMark, Bio-Rad, United States). The difference in absorbance at 450 nm and 650 nm was calculated. The absorbance at 450 nm and 650 nm in the LB liquid medium including no *E coli* was considered as a blank. The blank was subtracted from the difference in absorbance of the LB liquid medium with *E coli*. The value was used as the relative amount of *E coli*. Hundred percent of relative amount of *E coli* means confluent growth and 0% means no *E coli* in the LB liquid medium. Low value means high antibacterial effects of wound dressings.

### Bacterial retentivity of wound dressings

#### Preparation of different wound dressings

In this experiment, we compared the bacterial retentivity of wound dressings that have no antibacterial constituent. Some wound dressings possibly have a potency of keeping absorbed bacteria inside the wound dressings and preventing absorbed bacteria from leaking out the wound dressings as bacterial retentivity. The bacterial retentivity is also one of antibacterial effects because the bacterial retentivity contributes to reduction of bacteria on the wound surface, even if the wound dressings have no antibacterial constituent. Massive leakage of absorbed bacteria means low potency of the bacterial retentivity. Then, we prepared gauze, aluminum-coated viscose rayon (Metalline, Iwaki Kizai, Japan), hydropolymer (Tielle, Johnson & Johnson, New Jersy), polyurethane foams (Hydrosite, Smith & Nephew Medical Limited, United Kingdom), and hydrofiber (Aquacel, ConvaTec, USA). No wound dressing was regarded as a control. Next, each wound dressing or gauze was cut into squares 1 cm^2^ in size. Each piece of wound dressings was put in a well of a 24-well plate.

#### Preparation of bacteria

**Figure 1:**
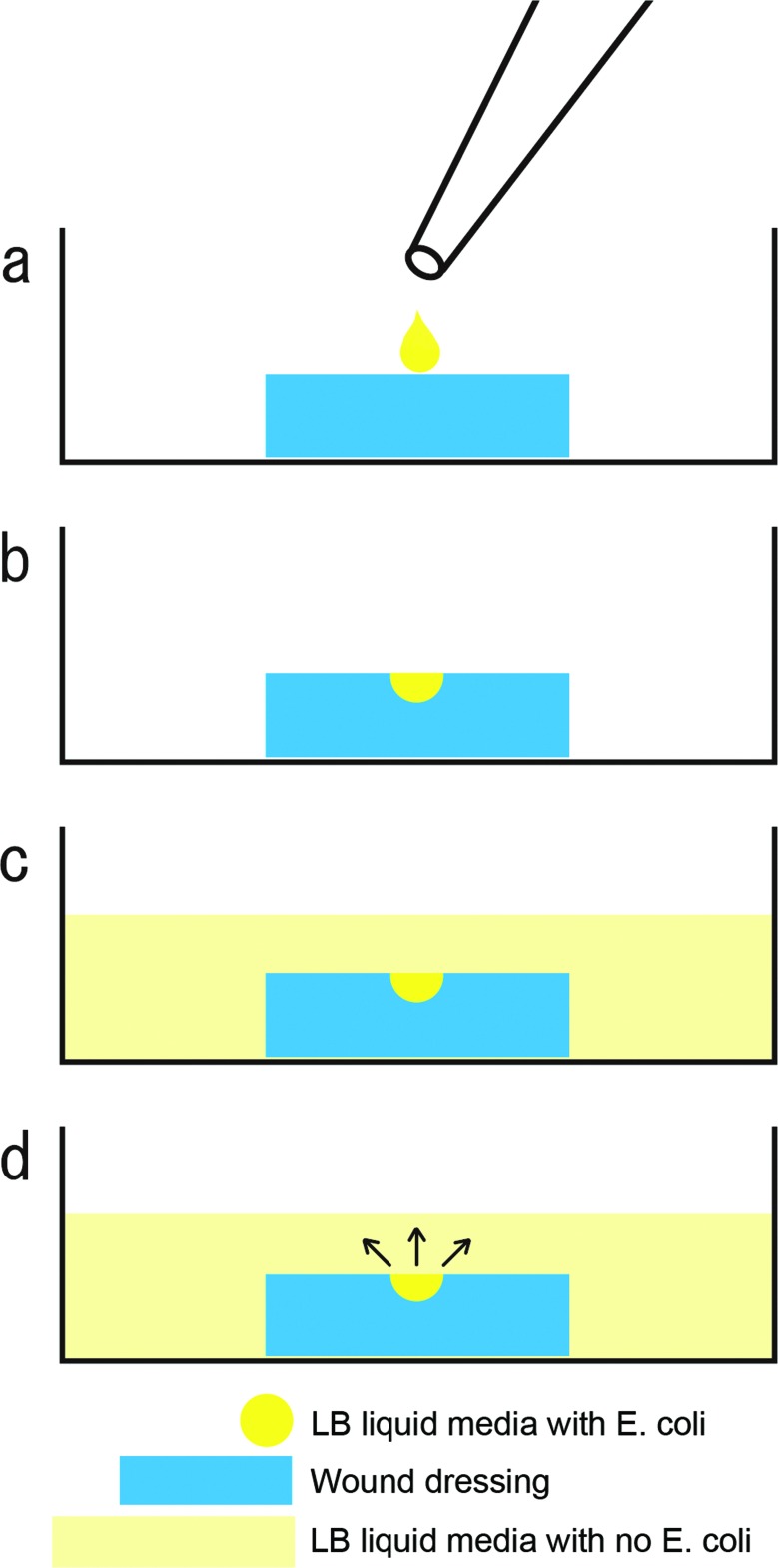
Diagrams showing the experimental procedure of bacterial retentivity of wound dressings. (*a*) 10 μL of the LB liquid medium with *Escherichia coli* was gently dropped on the superior surface of each wound dressing. (*b*) All wound dressings completely absorbed the drop of the LB liquid medium with *E coli* 10 minutes after the drop. (*c*) 2 mL of the antiseptic liquid medium with no *E coli* was gently poured in a well. (*d*) The black arrow indicates leakage of absorbed bacteria out the wound dressings. Massive leakage of absorbed bacteria means low bacterial retentivity. LB indicates Luria-Bertani.

We used nonpathogenic *E coli* to investigate the bacterial retentivity of wound dressings. *E coli* was cultured with the LB medium at 37°C until confluent growth and diluted to 1% with the antiseptic LB liquid medium. Next, 10 μL of the diluted LB liquid medium with *E coli* was gently dropped in the center of each wound dressing. The drop was completely absorbed in the wound dressing 10 minutes later. Then, 2 ml of the antiseptic LB liquid medium was gently poured in a well with the wound dressings that absorbed *E coli* (Fig 1). *E coli* in the 24-well plate was cultured in an incubator at 37°C for 24 hours.

#### Determination of the bacterial amount

Initially, 100 μL of the LB liquid medium with *E coli* was collected from each well with wound dressings at 1, 5, 15, 30 minutes and 1, 3, 6, 12, 24 hours after starting the culture and poured in a 96-well microplate. Then, 10 μL of cell proliferation reagent WST-1 was added to each well of 96-well microplate and mixed slightly. The LB liquid medium with WST-1 was incubated at 37°C for 2 hours. Next, absorbance at 450 nm and 650 nm was determined. The difference in absorbance at 450 nm and 650 nm was calculated. The absorbance at 450 nm and 650 nm in the LB liquid medium including no *E coli* was considered as a blank. The blank was subtracted from the difference in absorbance of the LB liquid medium with *E coli*. The value was used as the relative amount of *E coli*. Hundred percent of relative amount of *E coli* means confluent growth and 0% means no *E coli* in the LB liquid medium. Low value means high retentivity of wound dressings.

#### Statistical analysis

Statistical analysis was conducted with the Tukey-Kramer method. Significant differences were confirmed at *P* < 0.05. All the data are presented as means. The total (n) in each group was 3.

## RESULTS

### Antibacterial effects of wound dressings

**Figure 2:**
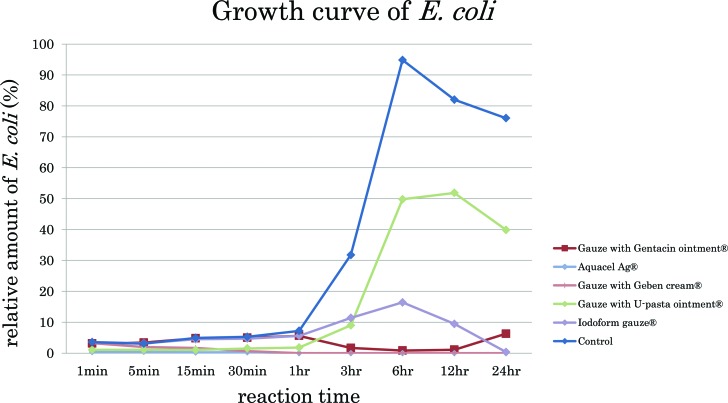
A line graphs shows the antibacterial effects in the groups of gauze with Gentacin ointment, Aquacel Ag, gauze with Geben cream, gauze with U-pasta ointment, iodoform gauze, and control. Hundred percent of relative amount of *E coli* means confluent growth and 0% means no *E coli* in the LB liquid medium. High amount of *E coli* means that the wound dressings have a low antibacterial effect. In contrast, low amount of *E coli* means that the wound dressings have a high antibacterial effect. LB indicates Luria-Bertani.

The growth curves of *E coli* in each group were different (Fig 2, Table 1). In these line graphs, 100% means confluent growth of *E coli* and 0% means no growth. In the control group, *E coli* rapidly increased 3 hours after starting and reached confluent growth 6 hours after starting. Aquacel Ag and U-pasta ointment exerted an antibacterial effect immediately after the start. This means that the antibacterial constituent of Aquacel Ag and U-pasta ointment dissolved in the LB liquid medium immediately after the start. Then, Aquacel Ag significantly preserved the strong antibacterial effectiveness for 24 hours from the start. In the group of U-pasta ointment, *E coli* gradually increased and reached up to 40% of confluent growth 24 hours after the start. Gentacin ointment exerted an antibacterial effect 3 hours after the start and significantly preserved the strong antibacterial effectiveness for 24 hours from the start. Geben cream exerted a strong antibacterial effect 5 minutes after the start and significantly preserved the antibacterial effectiveness for 24 hours. Iodoform gauze exerted an antibacterial effect 3 hours after the start and subsequently maintained the strong antibacterial effectiveness.

The proliferation curves were of 2 types. In the first type of proliferation curves, *E coli* in the groups of Gentacin ointment, U-pasta ointment, iodoform gauze initially proliferated to some extent and decreased gradually 3 to 6 hours after the start. In the second type of proliferation curves, Aquacel Ag and Geben cream exerted a strong antibacterial effect from the start and inhibited proliferation of *E coli* until 24 hours after the start. The differences in antibacterial effectiveness among all groups were attributed to the differences of the antibacterial constituent.

### Bacterial retentivity of wound dressings

**Figure 3:**
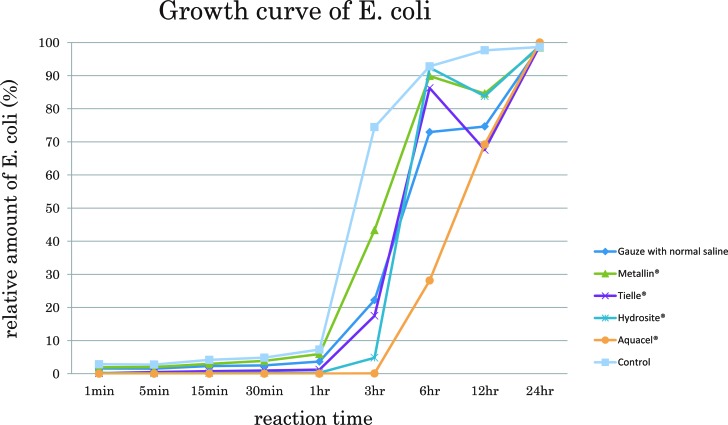
A line graph showing the results of bacterial retentivity of wound dressings. Hundred percent of relative amount of *Escherichia coli* means confluent growth and 0% means no *E coli* in the LB liquid medium. High amount of *E coli* means that the wound dressings have a low potency of bacterial retentivity. In contrast, low amount of *E coli* means that the wound dressings have a high potency of bacterial retentivity. LB indicates Luria-Bertani.

**Figure 4:**
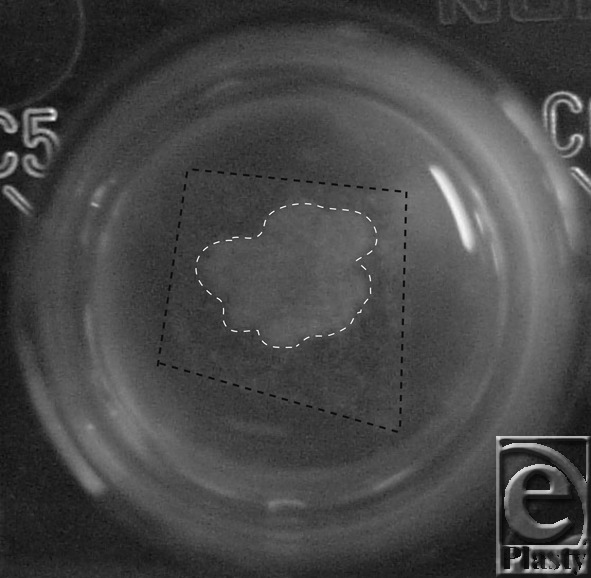
Gelling of Aquacel including the absorbed *Escherichia coli*. The black dot line shows the contour of Aquacel. The white dot line shows gelling of the absorbed LB liquid medium with *E coli*. LB indicates Luria-Bertani.

The bacterial retentivity of wound dressings was investigated using the wound dressings that had no antibacterial effectiveness (Fig 3, Table 1). In these line graphs, 100% means confluent growth of *E coli* and 0% means no growth. In the control group, *E coli* rapidly reached confluent growth 6 hours after the start. In the group of gauze and Metalline, *E coli* rapidly proliferated matching the proliferation curve of the control group, suggesting that gauze and Metalline had no bacterial retentivity. Next, in the groups of Tielle, Hydrosite, and Aquacel, no *E coli* was significantly detected in the LB liquid until 1 hour after the start, indicating that these wound dressings kept *E coli* inside the wound dressings and prevented them from leaking out the wound dressings. In the groups of Tielle and Hydrosite, *E coli* rapidly proliferated 3 hours after the start. Aquacel significantly kept *E coli* inside wound dressing until 3 hours after the start. In the group of Aquacel, *E coli* rapidly proliferated 6 hours after the start. Aquacel had a potency of gelling the absorbed liquid, resulting in a strong bacterial retentivity of Aquacel (Fig 4). The results demonstrated that some wound dressings had an effect like antibacterial effectiveness by keeping bacteria inside the wound dressings, although the wound dressings included no antibacterial constituent.

## DISCUSSION

Research of wound healing has contributed to the development of wound dressing materials, which provide optimal environment for wound healing. Almost all of the wound dressings are developed to maintain moist environment on the wound surface.^2^^,^^3^ On the contrary, humidity, warmth, and nutrient-rich soil are also good for bacterial proliferation.^8^ Therefore, wound dressings that maintain moist environment can desirably contribute to bacterial proliferation, not only the healthy cells in the wound, leading to wound infection. Nowadays, many wound dressings with antibacterial effects have been developed. Nonetheless, clinicians are often confused on selecting from a large variety of wound dressings. Then, many comparative studies on the antibacterial effects of wound dressings have been conducted.^11^^-^15 However, the antibacterial effects of wound dressings have not been investigated using the LB liquid medium in the *in vitro* model with *E coli* in detail. The bacterial retentivity of wound dressings as an additional antibacterial effect also remain unknown in detail. Therefore, in the present study, the antibacterial effects and bacterial retentivity of wound dressings were investigated to help clinicians easily select the optimal wound dressings for each wound.

The results indicated that Gentacin ointment, Aquacel Ag, Geben cream, U-pasta ointment, and iodoform gauze had antibacterial effects against *E coli*. Their antibacterial constituents were classified as antibiotic, silver ions and iodine. Gentamicin exerts antibacterial effectiveness by binding to the ribosomes of bacteria and inhibiting protein synthesis.^16^ Silver ions damage the bacterial cell membrane and inhibit protein synthesis by binding to the ribosomes through the cell membrane.^17^^,^^18^ Iodine denatures proteins by oxidation of the free iodine.^19^ These antibacterial constituents are stabilized as a chemical compound. Comparing silver ions of Aquacel Ag and Geben cream revealed that 1 parts per million of silver ions remain by substitution of sodium ions in the solution with silver ions in hydrofiber of Aquacel Ag. On the contrary, 10 mg of sulfadiazine silver exists in 1 g of Geben cream and exerts antibacterial effectiveness by dissolution into solution. In present study, Aquacel Ag exerted antibacterial effectiveness more quickly than Geben cream, suggesting that silver ions in Aquacel Ag dissolved in the LB liquid medium quicker than those in Geben cream, and that the antibacterial effects by silver ions were very strong in all wound dressings. Comparing iodine of U-pasta ointment with iodoform gauze revealed that 3 g of povidone-iodine (C_6_H_9_NO)n•xI existed in 100 g of U-pasta ointment and that 1.1 g of iodoform (CHI_3_) existed in 30 cm × 1 m of iodoform gauze. The differences of antibacterial strength, time of onset, and duration of the antibacterial effect were attributed to the differences of compound composition and content ratio of antibacterial constituents. The dissolution speed and inactivation time of antibacterial constituent were also thought to have some influences on the results. In the clinical practice, the conditions of wound surface—for example, moist environment, temperature, nourishment, and amount of exudate—depend on the patients, leading to differences in the antibacterial effects of the wound dressings. When the surface of the infected wound is viscous, the clinicians should select appropriate wound dressings for inhibition of infection because the antibacterial constituent exerts less antibacterial effect in such condition.

Next, the bacterial retentivity of wound dressings varied among the different types of wound dressings. Gauze and bonded material like Metalline retained little bacteria inside the wound dressing, resulting in bacterial proliferation outside the wound dressing. Hydropolymer of Tielle and polyurethane forms of Hydrosite had low retentivity. Tielle and Hydrosite are porous and have no potency of gelling. *E coli* may be absorbed in the porous cells and remain inside until 1 hour. On the contrary, hydrofibers of Aquacel absorbed exudate including bacteria and turned it into gel, leading to strong retentivity of bacteria inside the wound dressing. The retentivity of Aquacel was previously reported.^20^^,^^21^ Their studies indicated that carboxymethyl cellulose has a high bacterial retentivity by immobilizing bacteria with gelling. This retentivity also contributed to reduction of bacteria in the wound area. Therefore, the bacterial retentivity is one of the criteria to consider on selecting wound dressings.

The adverse effects of the antibacterial constituents include cytotoxicity, allergy, addiction, itching, and pain.^22^^-^25 The antibacterial constituents may damage the healthy cells, resulting in delayed wound healing. Long-term usage of antibacterial constituent beyond necessity may induce development of antibiotic-resistant bacteria and exacerbation of infection. Accordingly, wound dressings with strong antibacterial constituents are not always suitable for infected wounds. To achieve reduction of bacteria in an infected wound and development of granulation tissue in the wound, the wound dressings should be changed as necessary.

In the present study, the influences of immunity, humidity, and temperature of the wound surface were not examined. Also, antibacterial effects and bacterial retentivity against any bacteria except *E coli* should be investigated because infected wounds commonly include many types of bacteria, for example, *Staphylococcus aureus* and *Pseudomonas aeruginosa*. Therefore, the current in vitro results are not always applicable to clinical cases. However, in refractory cutaneous ulcers with poor blood flow or necrotic tissue, the present results may be applicable because the wound conditions are similar to those of the present study. The present study indicated the differences of antibacterial strength, time of onset, and duration of the antibacterial effect and bacterial retentivity between each wound dressing. Clinicians should use appropriate wound dressings according the wound condition in consideration of the different characteristics of wound dressings. It helps the clinicians select an optimal wound dressing from various types of wound dressings.

## Figures and Tables

**Table 1:** The results of antibacterial effects and bacterial retentivity of wound dressings. 100% means confluent growth of E. coli; 0% means no E. coli in the LB liquid medium. The total n in each group was 3

	1 min	5 min	15 min	30 min	1 h	3 h	6 h	12 h	24 h
Antibacterial effect of wound dressings									
Gauze with Gentacin ointment	3.1	3.4	4.8	5.0	5.6	1.7	0.8	1.1	6.3
Aquacel Ag	0.4	0.4	0.2	0.3	0.0	0.0	0.0	0.0	0.0
Gauze with Geben cream	3.2	2.0	1.7	0.6	0.0	0.0	0.0	0.0	0.0
Gauze with U-pasta ointment	1.1	1.1	1.0	1.5	1.8	9.0	49.8	51.8	39.9
Iodoform gauze	3.6	3.2	4.5	4.7	5.6	11.4	16.4	9.5	0.4
Control	3.5	3.1	4.9	5.3	7.2	31.8	94.8	82.0	76.0
Bacterial retentivity of wound dressings									
Gauze with normal saline	1.6	1.5	2.3	2.5	3.7	22.2	73.0	74.6	98.9
Metallin	1.9	2.0	3.0	3.9	6.0	43.4	89.9	84.6	98.5
Tielle	0.1	0.5	0.7	1.0	1.2	17.5	86.2	67.6	99.1
Hydrosite	0.3	0.1	0.2	0.3	0.3	4.8	92.4	83.8	99.0
Aquacel	0.0	0.0	0.0	0.0	0.0	0.1	28.1	69.2	100.0
Control	2.9	2.8	4.2	4.8	7.3	74.5	92.8	97.7	98.7
